# In-situ assessment of natural terrestrial-radioactivity from Uranium-238 (^238^U), Thorium-232 (^232^Th) and Potassium-40 (^40^K) in coastal urban-environment and its possible health implications

**DOI:** 10.1038/s41598-021-96516-z

**Published:** 2021-09-02

**Authors:** E. S. Joel, M. Omeje, O. C. Olawole, G. A. Adeyemi, A. Akinpelu, Z. Embong, M. A. Saeed

**Affiliations:** 1 Department of Earth Sciences, Anchor University, Lagos, Nigeria; 2 Department of Physics, Covenant University, Ota, Nigeria; 3 Department of Civil Engineering, Covenant University, Ota, Nigeria; 4 Faculty of Applied Science and Technology (FAST), Universiti Tun Hussein Onn Malaysia (UTHM), Pagoh Campus, Km 1, Jalan Panchor, 84600 Muar, Johor Malaysia; 5 Division of Science and Technology, University of Education Township, Lahore, Pakistan

**Keywords:** Cancer, Environmental sciences, Solid Earth sciences, Risk factors, Physics

## Abstract

The risk of natural terrestrial radioactivity on human health is often underestimated, and environmental safety awareness is necessary. Hence, this study aims to assess natural sources of gamma radiation emitter in coastal urban-environment using the radiometric technique. The dosage of gamma radiation from a parent radionuclide such as Uranium-238 (^238^U), Thorium-232 (^232^Th) and Potassium-40 (^40^K) and were measured using portable gamma spectroscopy. The result showed that the measured value of ^238^U activity was between 10.81 $\pm$ 0.69 and 46.31 $\pm$ 1.43 Bqkg^−1^. The mean value was estimated to be 35.44 $\pm$ 0.97 Bqkg^−1^ which is slightly higher than the world average. Meanwhile, ^232^Th activity ranges from 28.42 $\pm$ 1.12 to 69.43 $\pm$ 1.76 Bqkg^−1^ with the calculated mean value of 92.57 $\pm$ 1.17 Bqkg^−1^ while ^40^K activity ranged between 31.30 ± 1.32 and 328.65 ± 2.32 Bqkg^−1^ with the estimated mean 137.59 $\pm$ 2.42 Bqkg^−1^. Radiological parameters such as radium equivalent (R_eq_), internal hazard (H_int_) and external hazard (H_ext_) assessment were in the range of 66.00 Bqkg^−1^ to 141.76 Bqkg^−1^, 0.232 to 0.452 and 0.178 to 0.383, respectively. The measured values of gamma dose-rates ranged between 54.283 ± 0.78 and 117.531 ± 1.14 nGyh^−1^ with the calculated mean value of 84.770 ± 0.97 nGyh^−1^.

## Introduction

The natural habitat of ionising radiation was found in the eighteenth century, originating from radioactivity in groundwater, soils, rocks, and waterways, which are ecological materials^1–3^. It has been ascertained that radioactivity exists in its natural form on earth. About 82 per cent of humans have been said to be exposed and absorbed these emission doses^1^; however, this arises from the natural origin, which includes terrestrial-bodies, cosmic and exposition to these emission sources either as a result of inhaling of such^4^ can be detrimental to the human system. Several decades ago, multitudes of global investigations on radiation emanating from the subsurface were carried out^1^. The investigation report stated the various effects of background radiation on human health^1,5–7^.

Furthermore, gamma-emission, which originates from natural sources, is a result of primordial radionuclides, which are mainly Thorium-232 (^232^Th) and Uranium-238 (^238^U) series and their bye-decay products, and likewise, Potassium-40 (^40^K), which occurs as a trace-element in the earth's subsurface. These occurring natural radionuclides are dependent on the local geology of each area in the world^4^. Some quarries and springs add to the dose rate of radiation emitting from the subsurface in some regions of the earth, which are known to be high^6^.

The radionuclides that can be found in the environment are more than sixty (60). These are grouped into three (3) categories, namely cosmogenic (which occurs as a result of the interaction of rays from cosmic bodies), anthropogenic activities (occur through human technological development), and primordial (which exist before the creation of earth). Radionuclides live naturally in food, soil, water, air, oceans, and building houses' building materials^8,9^.

In addition, close to 50 per cent of natural radiation exposure that people are exposed to is connected with radon gas^6^. One source that causes cancer for the patients is traumatising from gastrointestinal and respiratory system hiccup^6^. The portion of radon which originates from radionuclides enters the human body system through breathing and drinking water. The aerosols tend to be the deposit source in the lungs where radiation is ejected and reported to be the likely cause of lung cancer^6–8^. Besides the radiation effect that emanates from the soils due to population exposure that uses grounds as a building material, the human body can also be affected by taking food consisting of radionuclide as a contaminant through the food chain from deep soil layers^10–20^.

Consequently, it has been noted that the level of terrestrially environmental-radiation in its specification is interconnected to the composition of the geology of an area^1,8,9^. Each lithological separated location contributes majorly to the subsurface radiation. The most coastal environment has been adjudged to be economically beneficial due to the natural deposits such as beaches, river bodies, natural sand (used for building purposes), and other mineral resources within such environments^8,9^. As a result, the human population usually migrates to this environment in large numbers for their sustenance. Therefore, it is necessary to ascertain how safe this environment is in radiation safety since radiation occurs naturally. Hence, the research focuses on the radionuclide existence in coastal urban area, mainly Uranium, Thorium and Potassium. Because these radionuclides are primarily found in the region's soil, where people live and work, whether because of the food chain or their homes, if these radionuclides are above the legal limit, it's bound to affect the health of the residents and even cause lung cancer. In addition, this is to ascertain the level of radiation in the area and evaluate the health impacts of such radiation on people settling in such a coastal area. Also, to establish the possible source of radioactive concentration in the study area if it connects with the natural deposit of kaolin and gypsum. The study, therefore, will serve as a baseline for house building contractors and residents of such coastal location on possible hotspot area of such radionuclides before embarking on any building project.

## Methodology

### Geological description of the sampling area

**Figure 1:**
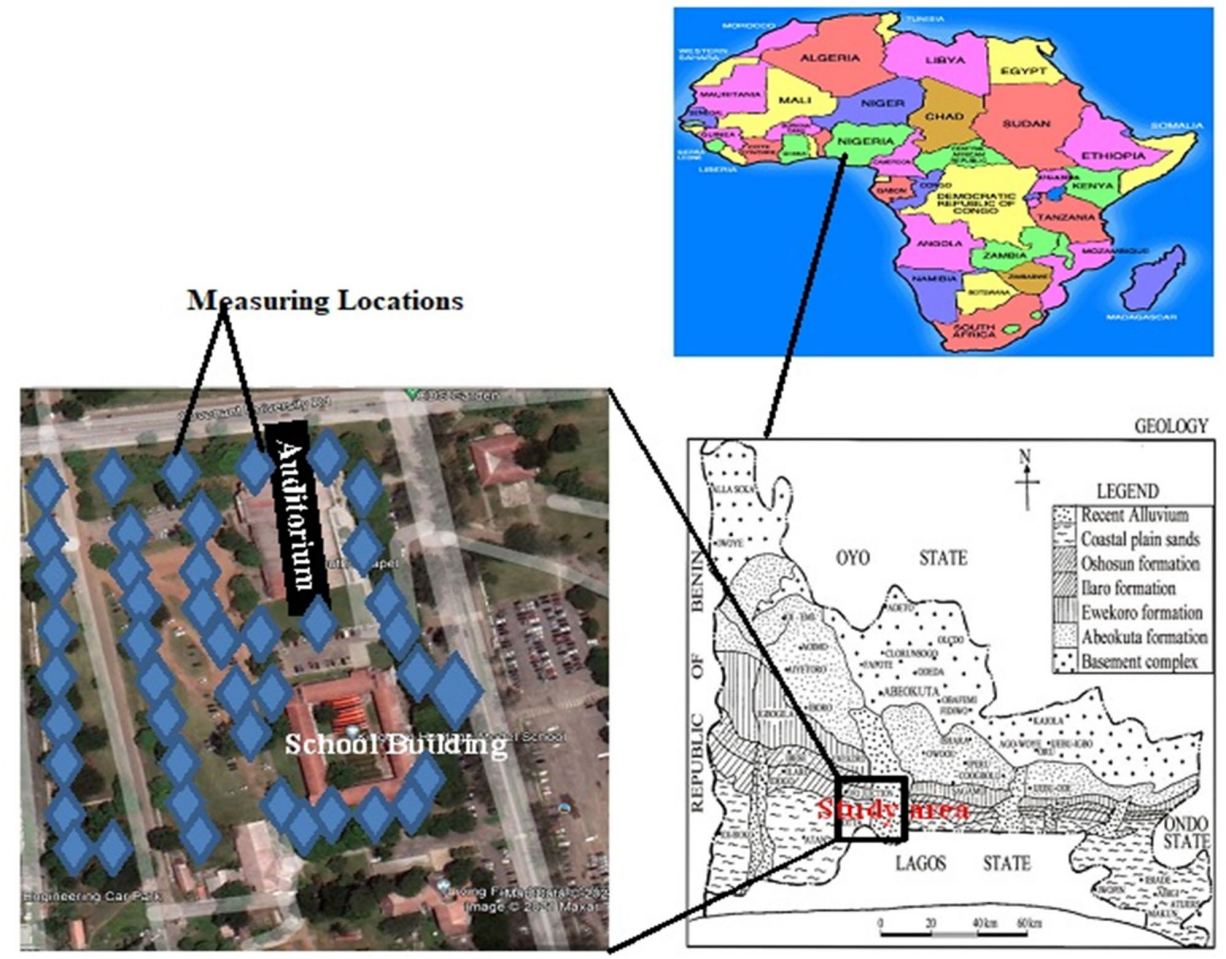
Base map of the measuring area (ArcGIS 10.8.1; https://desktop.arcgis.com/en/license-manager/2020.1/arcgis-license-manager).

The study region is generally a gently sloping low-lying area. It falls within the eastern Dahomey (or Benin) Basin of southwestern Nigeria, stretching along the Gulf of Guinea's continental margin. The study area's local geology lies within the sedimentary rock sequence of Dahomey Basin, which extends from the eastern part of Ghana through Togo and Benin Republic to the western margin of the Niger Delta^21,22^ (Fig. 1). The local geology's sequence arrangement that underlain the study area is as follows: Recent Alluvium (Quaternary age), which trends towards the south-east and central part of the study area and formed a boundary with Coastal Plain Sands in the west. This formation is followed by Coastal Plain Sands (Tertiary age—Pliocene), which is located in the west, southwest, and eastern part of the study area and also formed the boundary with the Ilaro Formation in the northwest. Ilaro Formation (Tertiary age—Eocene) overlaid both Coastal Plain Sands and Recent Alluvium and created the border with Ewekoro Formation/OshosunFormation/Akinbo Formation. This formation cuts across the northwest to the northeast of the study area. This geological formation is followed by Ewekoro Formation/Oshosun Formation/Akinbo Formation (Cretaceous—Paleocene). It cuts across north–north to northeast trend. The last geological formation that underlies Ewekoro Formation is Abeokuta Formation, Cretaceous age (Senonia). This formation formed a boundary with the Basement Complex in the North Ewekoro Formation in the northeast^23^.

The Dahomey basin comprises the Ogun River and Owena basin. The basin's tectonic structure is simple, forming a monocline against the basement outcrop to the North, with only a little evidence of faulting^24^. The area is characterised by two major climatic seasons: dry season spanning from November to March and rainy (or wet) season between April and October. Occasional rainfalls are usually witnessed within the dry season, particularly along the region adjoining the coast. Mean annual rainfall is more significant than 2000 mm and forms the primary groundwater source in the area.

### Instrumentation

The instrument used to measure gamma-dose rates and the emitted radionuclides used in this study is the portable handheld radiation detector (Super-SPEC RS 125). This instrument works based on the principle of radioactivity, which is the spontaneous disintegration of radioactive elements with the emission of gamma radiation and other particles such as alpha and beta. The equipment has a high degree of accuracy with probable measurement errors of about 5% of measured radionuclides concentration. The portable equipment came with an integrated design and a large detector, direct assay read-out, storage data point, full of weather protection, easily used, and highly sensitive. The count display of RS-125 Super-SPEC on the front side of the panel in cps at 1/sec update rate. The variable of the SCAN mode of RS-125 Super SPEC usually stores data in the device's memory through Bluetooth connection to external storage of the handheld device. The data's location is gotten through the External Global Positioning System (GPS) connection to the data stream via Bluetooth connection to the device. RS-125 Super SPEC provides the analysis of sample concentration and directly displayed the radionuclides, namely Potassium—^40^K (%), Uranium—^238^U (ppm), and Thorium—^232^Th (ppm). It also has a user-selectable sample time for optimum analysis. The RS-125 Super SPEC comes with utility software used for downloads of the stored data in memory. All the data in the memory of the handheld Super SPEC (RS-125) device can be transferred to the personal computer through Bluetooth or USB. Its operation does not require sources of radioactive content, and it was manufactured by an independent private company called Radiation-Solutions Incorporation situated at 386, Watline Avenue, Mississauga in Ontario, Canada)^24^. The calibration of the handheld radiation detector was done according to the guidelines of IAEA-Tecdoc before use. This procedure starts the arrangement between the measured radionuclides' counts (Thorium, Potassium, and Uranium). This approach allows the spectro-meter to decide on subjective ascertainment of Uranium, Thorium, and Potassium make-up of soils, environmental- wastes, and surface- rocks. The calibration of both ground and airborne radiation instruments is done based on the global quality invented by Canada's Geological Survey (GSC) traceable^25^. The portable instrument's calibration was done to ensure consistency and accuracy in estimating Potassium, Uranium, and Thorium. The impact of this radiation in the atmosphere could be demonstrated due to the rate of exposure rate or dose rate absorbed by the use of conversion factors emanating through radio-element concentrating in the samples to be measured. The measured data can be converted using the following conversion factor: For Uranium-238, 1 ppm = 12.35 Bqkg^−1^, Thorium-232, 1 ppm = 4.06 Bqkg^−1^ and Potassium-40, 1% = 313 Bqkg^−1^.

The activity concentrations were calculated using Eq. (1)^26,27^.

1 $${C}_{s}={C}_{ref}\frac{\frac{{P}_{s}}{{D}_{s}}-\frac{{P}_{b}}{{D}_{b}}}{\left(\frac{{P}_{ref}}{{D}_{ref}}-\frac{{P}_{b}}{{D}_{b}}\right) {M}_{s}}$$

where C_s_ and C_ref_ are the activity concentrations in Bq/kg of the measured data. P_s_, P_ref_ and P_b_ are the photopeak areas, standard reference materials and the background photopeak gamma lines, respectively, which is dimensionless. Also, D_s_, D_ref_ and D_b_ are the counting duration/time in seconds, standard reference materials, and background.

**Figure 2:**
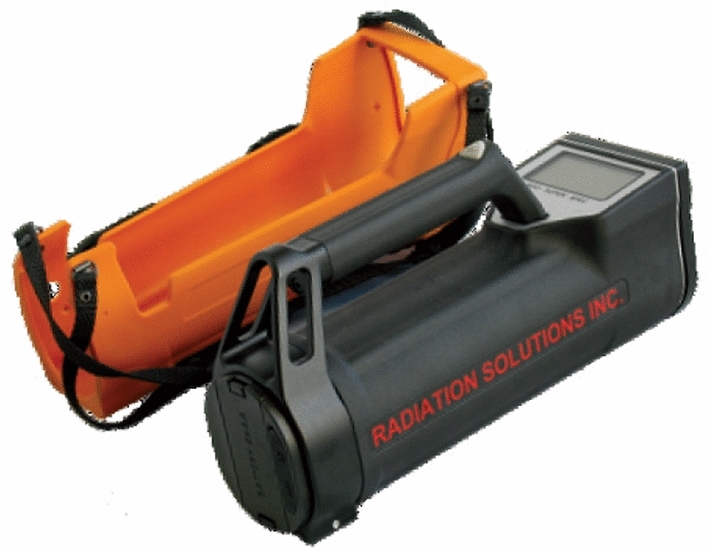
In-situ equipment RS-125 with the following specifications: Large NaI(Tl) crystal: 5 × 5 cm (2 × 2 in.), 1024 Channel spectrometer and Energy Range of 30–3000 keV.

The radiation detector (Fig. 2) was held 1 m above the ground at every measurement point; readings were taken four times at every station, and their average was calculated to ensure accuracy. In addition, the GPS coordinates were noted at each station, and readings were taken at an interval of 90 s at each measuring location. The instrument's reading was in parts per million (ppm); the mean results were obtained and converted to Becquerel per kilogram (Bqkg^−1^).

### Estimation of radiological parameters

The radiological parameters indices were determined from the measured data. These radiological parameters used in this present study include radium equivalent, internal-radiation hazard index, and external-radiation hazard index. These parameters have been established based on equations reported by^1^ and have been used by various researchers^8,28–33^, which have proved the reliability of these equations.

#### Radium equivalent radiological factor, (R_eq_)

The radium equivalent radiological factor refers to the frequent denominator used to compare radionuclides present in any material, which has been applied in this study to compare the radionuclides concentrations measured from the subsurface. Radium equivalent activities were evaluated based on Uranium-238, Thorium-232, and Potassium-40 at typical values of 370, 259, and 4810 Bqkg^−1^. Equation (2)^1,8^ was used in the estimation of the radium-equivalent activity.

2 $${R}_{eq}= {C}_{U}+1.43 {C}_{Th}+0.077 {C}_{K}$$

where C_U_, C_Th,_ and C_K_ are activity concentrations of ^238^U, ^232^Th, and ^40^K, respectively, in Bqkg^−1^.

#### Internal hazard assessment (H_int_)

The hazard which is defined by H_in_ and can be determined using Eq. (2)^4,8^:

3 $$ {\text{H}}_{{{\text{in}}}} = \left( {{\text{C}}_{{\text{U}}} /{185}} \right) + \left( {{\text{C}}_{{{\text{Th}}}} /{259}} \right) + \left( {{\text{C}}_{{\text{K}}} /{481}0} \right) $$

#### External hazard assessment (H_ext_)

The estimation of external risk assessment (H_ex_) associated with gamma dose rays emanating from the subsurface was done by applying Eq. (4) as used by^1^ and^8^**.**

Where C_U_, C_Th_, and C_K_ are the concentrations of activities in Bkg^−1^.

4 $$ {\text{H}}_{{{\text{ex}}}} = {\text{ C}}_{{\text{U}}} /{37}0 \, + {\text{ C}}_{{{\text{Th}}}} /{259 } + {\text{ C}}_{{\text{K}}} /{481}0 $$

## Results and discussions

### Measured naturally-occurring radiation (MNOR)

**Figure 3:**
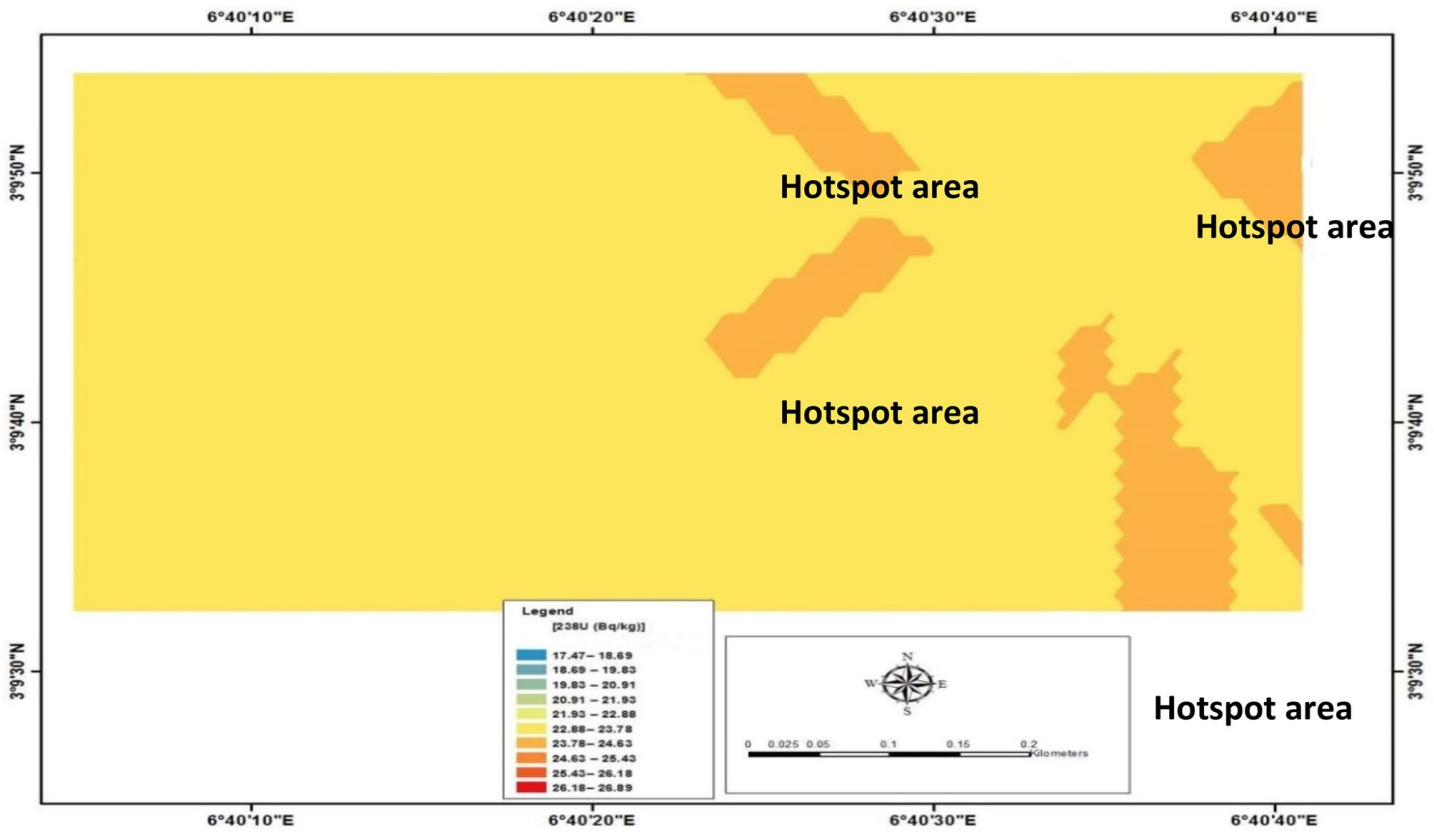
Showing the distribution pattern of Uranium-238 in the study area.

**Figure 4:**
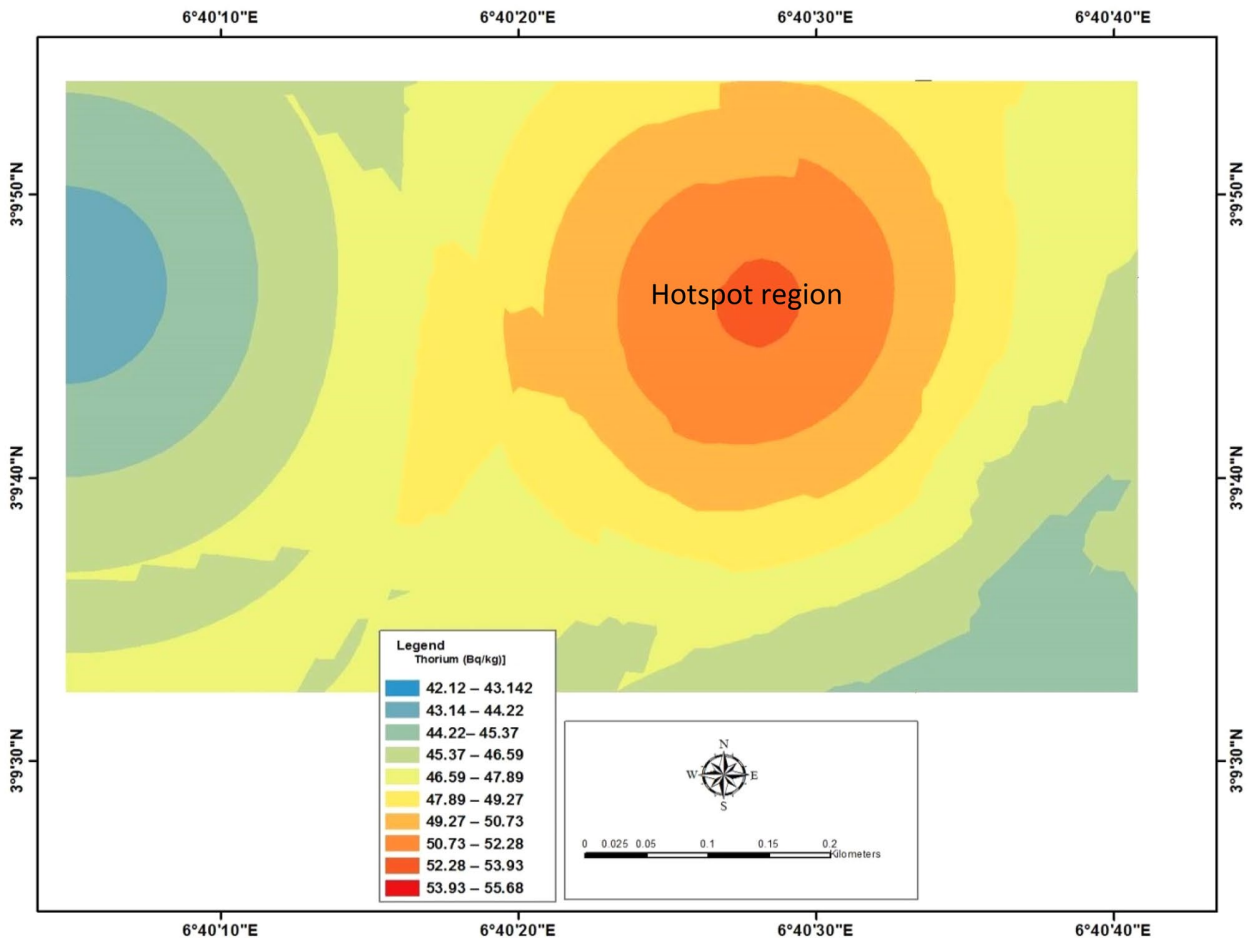
Showing the distribution pattern of Thorium-232 (^232^Th) in the study area.

**Figure 5:**
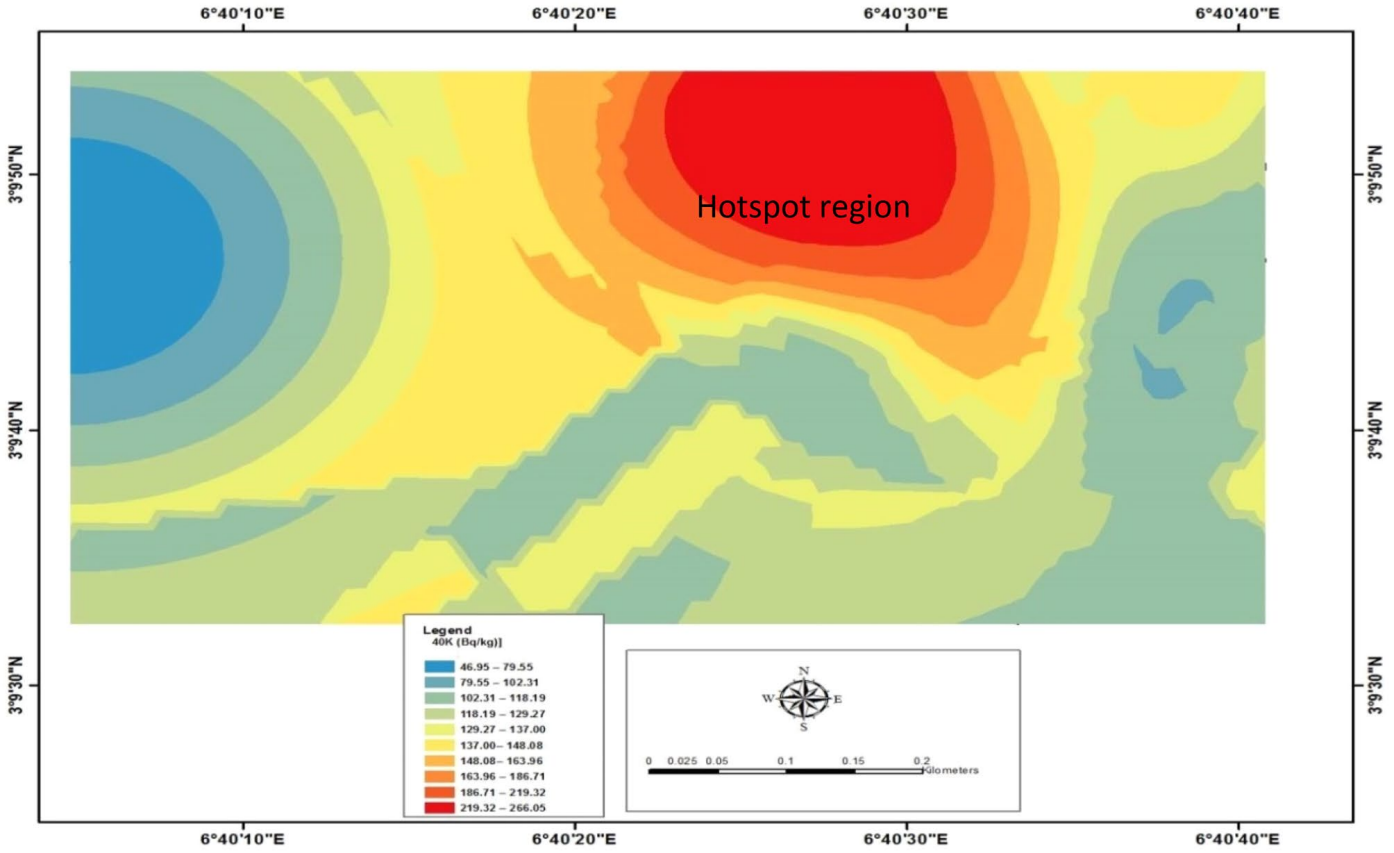
The distribution pattern of Potassium-40 (^40^K) in the study area.

The measured radionuclides and the distribution patterns for Uranium-238 (^238^U), Thorium-232 (^232^Th), and Potassium-40 (^40^K) *from 120 sampling points* in the study area are shown in Table 1, Figs. 3, 4, and 5, respectively. Table 1 showed the concentration of radionuclides measured across the study area. The radioactivity concentration ranged between 10.81 $\pm$ 0.69 Bqkg^−1^ and 46.31 $\pm$ 1.43 Bqkg^−1^. It was observed that the highest value of 46.31 $\pm$ 1.43 Bqkg^−1^ of Uranium-238 measured was noted at measuring location 71, while the lowest value of 10.81 $\pm$ 0.69 Bqkg^−1^ was reported at measuring area 31. The mean value was estimated to be 35.44 $\pm$ 0.97 Bqkg^−1^ compared with the world average of 33 Bqkg^−1^^1^. The standard deviation was also calculated to be 6.57 $\pm$ 0.59 Bqkg^−1^. The observation showed that the estimated mean value of uranium was high when compared with the world average. This occurrence might have been due to anthropogenic activities such as construction activity, which involves constructing materials such as cement, sand, and other imported decorative materials buried in the subsurface after usage. The distribution pattern of uranium concentration in the study area is shown in Fig. 3, with a hotspot indicated in the eastern part of the study area selected for this investigation. In addition, in observing Fig. 3, it was observed that the hotspot region for the Uranium-238 concentration was noted in the central, northeastern and southeastern of the study area.

**Table 1:** The measurement of ^238^U, ^232^Th and ^40^K radioactivity surface rock at the study area.

Measuring locations	^238^U (Bqkg^−1^)	^232^Th (Bqkg^−1^)	^40^K (Bqkg^−1^)
1	22.54 $\pm$ 1.00	38.06 $\pm$ 1.16	133.03 $\pm$ 2.43
2	24.08 $\pm$ 1.03	40.60 $\pm$ 1.34	54.78 $\pm$ 1.56
3	11.12 $\pm$ 0.70	43.95 $\pm$ 1.40	78.25 $\pm$ 1.86
4	16.06 $\pm$ 0.84	42.93 $\pm$ 1.38	179.98 $\pm$ 2.83
5	28.10 $\pm$ 1.12	45.17 $\pm$ 1.42	54.78 $\pm$ 1.56
6	23.77 $\pm$ 1.03	42.12 $\pm$ 1.37	46.95 $\pm$ 1.44
7	32.11 $\pm$ 1.19	42.83 $\pm$ 1.38	70.43 $\pm$ 1.77
8	28.41 $\pm$ 1.12	44.25 $\pm$ 1.40	86.08 $\pm$ 1.96
9	28.10 $\pm$ 1.12	33.60 $\pm$ 1.22	46.95 $\pm$ 1.44
10	17.29 $\pm$ 0.87	44.15 $\pm$ 1.40	93.90 $\pm$ 2.04
11	15.13 $\pm$ 0.82	44.56 $\pm$ 1.41	148.68 $\pm$ 2.57
12	36.74 $\pm$ 1.28	54.51 $\pm$ 1.56	133.03 $\pm$ 2.43
13	14.51 $\pm$ 0.80	39.89 $\pm$ 1.33	140.85 $\pm$ 2.50
14	17.29 $\pm$ 0.87	45.98 $\pm$ 1.43	195.63 $\pm$ 2.95
15	17.91 $\pm$ 0.89	48.92 $\pm$ 1.47	93.90 $\pm$ 2.04
16	24.08 $\pm$ 1.03	53.99 $\pm$ 1.55	148.68 $\pm$ 2.57
17	22.54 $\pm$ 1.00	58.67 $\pm$ 1.61	211.27 $\pm$ 3.06
18	20.99 $\pm$ 0.97	52.17 $\pm$ 1.52	133.02 $\pm$ 2.43
19	25.01 $\pm$ 1.05	50.04 $\pm$ 1.49	54.77 $\pm$ 1.56
20	13.89 $\pm$ 0.79	47.20 $\pm$ 1.45	54.78 $\pm$ 1.56
21	14.82 $\pm$ 0.81	42.43 $\pm$ 1.37	46.95 $\pm$ 1.44
22	14.82 $\pm$ 0.81	36.54 $\pm$ 1.27	70.42 $\pm$ 1.77
23	20.07 $\pm$ 0.94	30.86 $\pm$ 1.17	23.48 $\pm$ 1.02
24	21.30 $\pm$ 0.97	44.25 $\pm$ 1.40	31.30 $\pm$ 1.18
25	30.26 $\pm$ 1.16	41.82 $\pm$ 1.36	31.31 $\pm$ 1.18
26	16.36 $\pm$ 0.85	47.09 $\pm$ 1.45	86.08 $\pm$ 1.96
27	28.09 $\pm$ 1.12	49.23 $\pm$ 1.48	140.85 $\pm$ 2.50
28	14.82 $\pm$ 0.81	57.75 $\pm$ 1.60	289.53 $\pm$ 3.59
29	19.76 $\pm$ 0.93	47.19 $\pm$ 1.45	172.15 $\pm$ 2.77
30	20.06 $\pm$ 0.94	52.47 $\pm$ 1.53	101.73 $\pm$ 2.13
31	10.80 $\pm$ 0.69	50.34 $\pm$ 1.50	266.05 $\pm$ 3.44
32	12.04 $\pm$ 0.73	47.40 $\pm$ 1.45	187.80 $\pm$ 2.89
33	19.45 $\pm$ 0.93	52.37 $\pm$ 1.53	70.43 $\pm$ 1.77
34	29.64 $\pm$ 1.15	41.21 $\pm$ 1.35	62.60 $\pm$ 1.67
35	21.92 $\pm$ 0.99	38.37 $\pm$ 1.31	148.68 $\pm$ 2.57
36	13.58 $\pm$ 0.78	57.55 $\pm$ 1.60	219.10 $\pm$ 3.12
37	23.46 $\pm$ 1.02	46.89 $\pm$ 1.44	125.20 $\pm$ 2.36
38	27.47 $\pm$ 1.11	42.63 $\pm$ 1.38	140.85 $\pm$ 2.50
39	29.33 $\pm$ 1.15	46.69 $\pm$ 1.44	164.33 $\pm$ 2.70
40	27.17 $\pm$ 1.09	44.56 $\pm$ 1.41	211.28 $\pm$ 3.06
41	14.51 $\pm$ 0.80	43.04 $\pm$ 1.38	172.15 $\pm$ 2.77
42	25.00 $\pm$ 1.05	43.65 $\pm$ 1.39	101.72 $\pm$ 2.13
43	25.62 $\pm$ 1.07	36.84 $\pm$ 1.28	54.78 $\pm$ 1.56
44	24.39 $\pm$ 1.04	36.54 $\pm$ 1.27	56.73 $\pm$ 1.59
45	18.21 $\pm$ 0.90	55.32 $\pm$ 1.57	54.77 $\pm$ 1.56
46	12.04 $\pm$ 0.73	43.95 $\pm$ 1.40	54.77 $\pm$ 1.56
47	27.17 $\pm$ 1.09	28.42 $\pm$ 1.12	46.95 $\pm$ 1.44
48	30.25 $\pm$ 1.16	37.05 $\pm$ 1.28	70.42 $\pm$ 1.77
49	22.23 $\pm$ 0.99	52.68 $\pm$ 1.53	86.07 $\pm$ 1.96
50	26.24 $\pm$ 1.08	52.78 $\pm$ 1.53	93.91 $\pm$ 2.04
51	30.87 $\pm$ 1.17	49.43 $\pm$ 1.48	187.8 $\pm$ 2.89
52	36.74 $\pm$ 1.28	46.89 $\pm$ 1.44	226.93 $\pm$ 3.18
53	28.09 $\pm$ 1.12	54.71 $\pm$ 1.56	266.05 $\pm$ 3.18
54	31.18 $\pm$ 1.18	49.84 $\pm$ 1.49	117.38 $\pm$ 2.28
55	12.35 $\pm$ 0.74	45.37 $\pm$ 1.42	195.63 $\pm$ 2.95
56	33.04 $\pm$ 1.21	43.74 $\pm$ 1.39	31.30 $\pm$ 1.18
57	30.57 $\pm$ 1.17	41.81 $\pm$ 1.36	62.60 $\pm$ 1.67
58	25.93 $\pm$ 1.07	44.56 $\pm$ 1.41	117.37 $\pm$ 2.28
59	20.07 $\pm$ 0.94	52.68 $\pm$ 1.53	328.65 $\pm$ 3.82
60	19.76 $\pm$ 0.94	46.18 $\pm$ 1.43	219.10 $\pm$ 3.12
61	26.55 $\pm$ 1.09	40.40 $\pm$ 1.34	297.35 $\pm$ 3.63
62	23.46 $\pm$ 1.02	50.45 $\pm$ 1.50	266.05 $\pm$ 3.45
63	28.72 $\pm$ 1.13	55.83 $\pm$ 1.58	250.40 $\pm$ 3.34
64	26.24 $\pm$ 1.08	43.54 $\pm$ 1.39	219.10 $\pm$ 3.12
65	14.20 $\pm$ 0.79	46.89 $\pm$ 1.44	219.11 $\pm$ 3.12
66	20.10 $\pm$ 0.97	41.41 $\pm$ 1.36	46.95 $\pm$ 1.44
67	25.94 $\pm$ 1.07	44.36 $\pm$ 1.40	117.38 $\pm$ 2.28
68	12.97 $\pm$ 0.76	52.48 $\pm$ 1.53	164.33 $\pm$ 2.70
69	14.82 $\pm$ 0.81	43.24 $\pm$ 1.39	39.13 $\pm$ 1.32
70	24.7 $\pm$ 1.05	46.39 $\pm$ 1.44	78.25 $\pm$ 1.86
71	46.31 $\pm$ 1.43	43.85 $\pm$ 1.40	140.85 $\pm$ 2.50
72	26.24 $\pm$ 1.08	42.73 $\pm$ 1.39	195.63 $\pm$ 2.95
73	24.70 $\pm$ 1.05	43.04 $\pm$ 1.38	211.28 $\pm$ 3.06
74	24.39 $\pm$ 1.04	53.49 $\pm$ 1.54	242.58 $\pm$ 3.28
75	16.67 $\pm$ 0.86	53.28 $\pm$ 1.54	148.68 $\pm$ 2.57
76	23.15 $\pm$ 1.01	47.71 $\pm$ 1.46	133.03 $\pm$ 2.43
77	20.38 $\pm$ 0.95	45.88 $\pm$ 1.43	179.98 $\pm$ 2.83
78	27.48 $\pm$ 1.11	49.53 $\pm$ 1.48	46.95 $\pm$ 1.44
79	16.67 $\pm$ 0.86	50.55 $\pm$ 1.50	101.73 $\pm$ 2.13
80	22.54 $\pm$ 1.00	49.33 $\pm$ 1.48	211.28 $\pm$ 3.06
81	23.77 $\pm$ 1.02	50.34 $\pm$ 1.50	203.45 $\pm$ 3.01
82	25.62 $\pm$ 1.07	43.75 $\pm$ 1.39	133.02 $\pm$ 2.43
83	25.01 $\pm$ 1.05	52.37 $\pm$ 1.53	133.01 $\pm$ 2.43
84	25.01 $\pm$ 1.05	52.27 $\pm$ 1.52	101.73 $\pm$ 2.13
85	26.86 $\pm$ 1.09	65.98 $\pm$ 1.71	164.33 $\pm$ 2.70
86	25.93 $\pm$ 1.07	56.23 $\pm$ 1.58	101.73 $\pm$ 2.13
87	32.11 $\pm$ 1.19	53.19 $\pm$ 1.54	164.34 $\pm$ 2.70
88	27.47 $\pm$ 1.11	52.68 $\pm$ 1.53	133.02 $\pm$ 2.43
89	26.24 $\pm$ 1.08	50.65 $\pm$ 1.50	101.71 $\pm$ 2.13
90	16.36 $\pm$ 0.85	46.89 $\pm$ 1.44	101.72 $\pm$ 2.13
91	21.92 $\pm$ 0.99	49.94 $\pm$ 1.49	39.13 $\pm$ 1.32
92	28.09 $\pm$ 1.12	41.21 $\pm$ 1.35	86.08 $\pm$ 1.96
93	24.7 $\pm$ 1.05	44.46 $\pm$ 1.41	148.68 $\pm$ 2.57
94	33.35 $\pm$ 1.22	54.70 $\pm$ 1.56	133.02 $\pm$ 2.43
95	27.17 $\pm$ 1.10	50.75 $\pm$ 1.50	140.85 $\pm$ 2.50
96	29.02 $\pm$ 1.14	46.99 $\pm$ 1.45	164.34 $\pm$ 2.70
97	25.63 $\pm$ 1.07	49.94 $\pm$ 1.50	133.02 $\pm$ 2.43
98	35.51 $\pm$ 1.26	50.45 $\pm$ 1.50	133.02 $\pm$ 2.43
99	33.96 $\pm$ 1.23	47.50 $\pm$ 1.45	133.03 $\pm$ 2.43
100	12.66 $\pm$ 0.75	48.31 $\pm$ 1.47	101.72 $\pm$ 2.13
101	28.09 $\pm$ 1.12	45.98 $\pm$ 1.43	219.10 $\pm$ 3.14
102	11.73 $\pm$ 0.72	39.59 $\pm$ 1.33	148.68 $\pm$ 2.57
103	24.39 $\pm$ 1.04	36.84 $\pm$ 1.28	117.37 $\pm$ 2.28
104	24.7 $\pm$ 1.05	32.78 $\pm$ 1.21	86.08 $\pm$ 1.96
105	20.07 $\pm$ 0.94	43.24 $\pm$ 1.39	187.80 $\pm$ 2.89
106	17.91 $\pm$ 0.89	50.65 $\pm$ 1.50	258.23 $\pm$ 3.39
107	20.07 $\pm$ 0.94	52.68 $\pm$ 1.53	109.55 $\pm$ 2.21
108	25.94 $\pm$ 1.07	58.36 $\pm$ 1.61	101.72 $\pm$ 2.13
109	25.32 $\pm$ 1.06	60.90 $\pm$ 1.65	187.80 $\pm$ 2.89
110	21.30 $\pm$ 0.97	56.94 $\pm$ 1.59	211.28 $\pm$ 3.06
111	27.48 $\pm$ 1.11	51.56 $\pm$ 1.51	219.10 $\pm$ 3.14
112	25.01 $\pm$ 1.05	69.43 $\pm$ 1.76	226.93 $\pm$ 3.18
113	34.89 $\pm$ 1.25	50.85 $\pm$ 1.50	133.02 $\pm$ 2.43
114	30.26 $\pm$ 1.16	39.99 $\pm$ 1.33	54.78 $\pm$ 1.56
115	20.07 $\pm$ 0.94	44.56 $\pm$ 1.41	125.20 $\pm$ 2.36
116	31.18 $\pm$ 1.18	48.42 $\pm$ 1.47	125.20 $\pm$ 2.36
117	14.20 $\pm$ 0.79	50.85 $\pm$ 1.50	172.15 $\pm$ 2.77
118	11.73 $\pm$ 0.72	62.73 $\pm$ 1.67	250.40 $\pm$ 3.34
119	19.76 $\pm$ 0.94	58.77 $\pm$ 1.62	226.93 $\pm$ 3.18
120	21.61 $\pm$ 0.98	61.51 $\pm$ 1.65	250.40 $\pm$ 3.34
Mean	35.44 $\pm$ 0.97	92.57 $\pm$ 1.17	137.59 $\pm$ 2.42
Standard deviation	6.57 $\pm$ 0.59	6.93 $\pm$ 0.63	70.10 $\pm$ 6.40

Table 1 showed the measured concentration of Thorium (^232^Th). It ranges from 28.42 $\pm$ 1.12 to 69.43 $\pm$1.76 Bqkg^−1^. The observed lowest value was noted at location 47, while the highest noticed at location 112 with the calculated mean value of 92.57 $\pm$ 1.17 Bqkg^−1^. Furthermore, the estimated mean value was compared with the world average of 45 Bq/kg. Therefore, it was noticed the estimated mean value was higher. The pattern distribution of ^232^Th concentration is shown in Fig. 4. Also, Thorium-232 (^232^Th) concentration increased from the western part of the study area to the eastern part, as shown in Fig. 4. Therefore, the hotspot region for the ^232^Th was noted at the eastern part of the study area. The high concentration of ^232^Th may result from anthropogenic materials' deposition, originating from a human-made source and industrial activities in the study area. This may have aggravated the radionuclides' natural existence in the study area. Therefore, structures with artificial natural ventilation such as air conditioner located in the eastern part of the study area should always be in 20% usage.

The variation of activity concentration of Potassium-40 (^40^K) is shown in Table 1. Potassium-40 (40K) measured the measured value between 31.30 ± 1.32 and 328.65 ± 2.32 Bqkg^−1^ with the estimated mean and standard deviation values of 137.59 $\pm$ 2.42 and 70.10 $\pm$ 6.40 Bqkg^−1^, respectively. The lowest measured Potassium-40 (^40^K) was noted in locations 24, 25, and 56, while the highest value was observed in 59. Furthermore, the decrease in Potassium-40 (^40^K) values was observed in both the study area's eastern and western parts. Besides, Fig. 5 showed the pattern of Potassium-40 (^40^K) distribution in the study area. The distribution of Potassium-40 (^40^K) radionuclide observed increased from western toward the northern part study area while decreasing the trend as approaching east of the study area. The hotspot region for Potassium-40 (^40^K) was observed at the central part of the study area, which flagged toward the North. However, comparing the average value with the world average of 420 Bqkg^−1^, the values were far below the recommended limit. Also, the three primordial radionuclides were compared; the results showed that Uranium-238 (^238^U) and Thorium-232 (^232^Th) are less in comparison with the Potassium-40 (^40^K). This is because Potassium-40 (^40^K) may be associated with the coarse organic-rich rocks, which are radioactive. Furthermore, the measured radionuclides were compared with a similar study by^34–36^.

### Estimated radiological parameters

#### Radium equivalent (Req)

Radium equivalent parameter is calculated using Eq. (1) with the standard measured values of 370, 259, and 4810 Bqkg^−1^. Table 2 showed the variation in radium equivalent for each measuring point. The estimated values ranged from 66.00 Bqkg^−1^ to 141.76 Bqkg^−1^, with the lowest and highest value noted at measuring points 23 and 112, respectively. The mean value is calculated to be 202.15 Bqkg^−1^. More elevated radium equivalent values were noticed across the study area though less than the recommended limit value of 370 Bqkg^−1^. Other measuring locations with high value of radium equivalent (˃ 100 Bqkg^−1^) include 12, 16, 17, 18, 27, 28, 29, 30, 31 etc. Also, a high concentration of radium equivalent noted in the study area is connected to the contribution of radionuclide (^238^U, ^232^Th and ^40^K) measured, particularly Potassium-40 (^40^K).

**Table 2:** The calculated value for a radiological parameter, Radium Equivalent, Inter Hazard index, External hazard index and Dose rate for all sampling location.

Measuring locations	Radium Equivalent (R_eq_) (Bqkg^−1^)	Inter Hazard Index (H_int_)	External Hazard Index (H_ext_)	Measured Dose Rate (D_rate_) (nGyh^−1^)
1	87.21	0.296	0.236	73.246 ± 0.90
2	86.36	0.298	0.233	71.198 ± 0.89
3	79.98	0.246	0.216	64.830 ± 0.85
4	91.31	0.290	0.247	76.397 ± 0.92
5	96.90	0.338	0.262	79.915 ± 0.94
6	87.62	0.301	0.237	71.963 ± 0.89
7	98.78	0.354	0.267	82.292 ± 0.96
8	98.32	0.342	0.266	81.698 ± 0.95
9	79.75	0.291	0.215	66.561 ± 0.86
10	87.66	0.283	0.237	71.987 ± 0.89
11	90.30	0.285	0.244	74.827 ± 0.91
12	124.93	0.437	0.337	104.400 ± 1.08
13	82.40	0.262	0.223	68.497 ± 0.87
14	98.10	0.312	0.265	82.134 ± 0.96
15	95.10	0.305	0.257	77.802 ± 0.92
16	112.75	0.370	0.304	93.448 ± 1.02
17	122.71	0.393	0.331	102.171 ± 1.07
18	105.85	0.343	0.286	87.346 ± 0.99
19	100.78	0.340	0.272	82.434 ± 0.96
20	85.60	0.269	0.231	69.082 ± 0.88
21	79.11	0.254	0.214	64.060 ± 0.84
22	72.49	0.236	0.196	59.462 ± 0.81
23	66.00	0.232	0.178	54.283 ± 0.78
24	86.99	0.293	0.235	70.783 ± 0.89
25	92.47	0.332	0.250	76.341 ± 0.92
26	90.34	0.288	0.244	73.746 ± 0.91
27	109.34	0.371	0.295	91.267 ± 1.01
28	119.71	0.363	0.323	100.325 ± 1.06
29	100.51	0.325	0.271	83.868 ± 0.97
30	102.94	0.332	0.278	84.324 ± 0.97
31	103.28	0.308	0.279	86.604 ± 0.98
32	94.28	0.287	0.255	78.243 ± 0.93
33	99.77	0.322	0.269	81.141 ± 0.95
34	93.39	0.332	0.252	77.607 ± 0.93
35	88.23	0.298	0.238	74.265 ± 0.91
36	112.75	0.341	0.304	93.332 ± 1.01
37	100.16	0.334	0.271	83.186 ± 0.97
38	99.29	0.342	0.268	83.441 ± 0.97
39	108.75	0.373	0.294	91.490 ± 1.01
40	107.16	0.363	0.289	90.913 ± 1.01
41	89.31	0.280	0.241	74.462 ± 0.91
42	95.25	0.325	0.257	79.156 ± 0.94
43	82.53	0.292	0.223	68.487 ± 0.87
44	76.64	0.273	0.207	62.634 ± 0.83
45	101.54	0.323	0.274	81.990 ± 0.95
46	79.11	0.246	0.214	63.804 ± 0.84
47	71.43	0.266	0.193	60.014 ± 0.82
48	88.66	0.321	0.239	74.223 ± 0.91
49	104.12	0.341	0.281	85.284 ± 0.97
50	108.95	0.365	0.294	89.714 ± 1.00
51	116.02	0.397	0.313	97.803 ± 1.04
52	121.27	0.427	0.328	103.538 ± 1.07
53	126.82	0.418	0.342	107.312 ± 1.09
54	111.49	0.385	0.301	92.899 ± 1.02
55	92.29	0.283	0.249	76.920 ± 0.92
56	98.00	0.354	0.265	81.019 ± 0.95
57	95.19	0.340	0.257	79.129 ± 0.94
58	98.69	0.337	0.267	82.266 ± 0.96
59	120.71	0.380	0.326	102.702 ± 1.07
60	102.67	0.331	0.277	86.508 ± 0.98
61	107.22	0.361	0.290	92.653 ± 1.01
62	116.09	0.377	0.314	98.362 ± 1.05
63	127.82	0.423	0.345	107.856 ± 1.09
64	105.38	0.356	0.285	89.570 ± 1.00
65	98.13	0.303	0.265	82.177 ± 0.96
66	83.83	0.283	0.226	68.625 ± 0.87
67	98.40	0.336	0.266	82.041 ± 0.95
68	100.66	0.307	0.272	82.799 ± 0.96
69	79.66	0.255	0.215	64.327 ± 0.85
70	97.06	0.329	0.262	80.008 ± 0.94
71	119.86	0.449	0.324	102.108 ± 1.07
72	102.41	0.348	0.277	86.800 ± 0.98
73	102.51	0.344	0.277	86.966 ± 0.98
74	119.56	0.389	0.323	100.686 ± 1.06
75	104.32	0.327	0.282	85.849 ± 0.98
76	101.62	0.337	0.274	84.421 ± 0.97
77	99.84	0.325	0.270	83.611 ± 0.96
78	101.92	0.350	0.275	83.522 ± 0.96
79	96.79	0.306	0.261	79.078 ± 0.94
80	109.35	0.356	0.295	91.899 ± 1.01
81	111.43	0.365	0.301	93.526 ± 1.02
82	98.42	0.335	0.266	82.339 ± 0.96
83	110.15	0.365	0.297	91.261 ± 1.01
84	107.59	0.358	0.291	88.646 ± 0.99
85	133.86	0.434	0.361	110.431 ± 1.11
86	114.18	0.380	0.308	93.852 ± 1.02
87	120.82	0.413	0.326	101.192 ± 1.06
88	113.05	0.380	0.305	93.869 ± 1.02
89	106.50	0.359	0.288	87.996 ± 0.99
90	91.25	0.290	0.246	74.775 ± 0.91
91	96.35	0.319	0.260	78.229 ± 0.93
92	93.65	0.329	0.253	78.064 ± 0.93
93	99.72	0.336	0.269	83.521 ± 0.96
94	121.82	0.419	0.329	101.499 ± 1.06
95	110.59	0.372	0.299	92.089 ± 1.01
96	108.88	0.372	0.294	91.541 ± 1.01
97	107.28	0.359	0.290	89.150 ± 1.00
98	117.89	0.414	0.318	98.798 ± 1.05
99	112.13	0.395	0.303	94.140 ± 1.02
100	89.58	0.276	0.242	72.929 ± 0.90
101	110.72	0.375	0.299	93.954 ± 1.02
102	79.79	0.247	0.215	66.231 ± 0.86
103	86.12	0.299	0.233	72.359 ± 0.90
104	78.21	0.278	0.211	65.673 ± 0.85
105	96.36	0.314	0.260	81.050 ± 0.95
106	110.22	0.346	0.298	92.846 ± 1.02
107	103.83	0.335	0.280	85.174 ± 0.97
108	117.23	0.387	0.317	96.197 ± 1.03
109	126.87	0.411	0.343	105.306 ± 1.08
110	118.99	0.379	0.321	99.137 ± 1.05
111	118.08	0.393	0.319	99.527 ± 1.05
112	141.76	0.450	0.383	117.531 ± 1.14
113	117.85	0.413	0.318	98.676 ± 1.05
114	91.66	0.329	0.248	76.209 ± 0.92
115	93.43	0.307	0.252	77.494 ± 0.93
116	110.06	0.382	0.297	91.962 ± 1.01
117	100.18	0.309	0.271	82.775 ± 0.96
118	120.71	0.358	0.326	99.826 ± 1.05
119	121.27	0.381	0.327	100.979 ± 1.06
120	128.85	0.406	0.348	107.575 ± 1.09
Mean	202.15	0.671	0.275	84.770 ± 0.97
Minimum	66.00	0.232	0.178	54.283 ± 0.78
Maximum	141.76	0.450	0.383	117.531 ± 1.14

#### Internal hazard index, (H_int_)

The estimation of the internal hazard index was done using Eq. (2), which is associated with the gamma dose rate measured in the study area, shown in Table 2. For the study area to be suitable for siting building structure for safety reasons, the estimated internal hazard index must be less than unity as recommended by^37,38^ and^1^. This present study observed that H_in_ ranged between 0.232 and 0.452, with a calculated mean of 0.671. The lowest and highest values of the internal hazard index were noted at measuring points 23 and 112.

#### External hazard index, (H_ext_)

Table 2 showed the estimated external hazard index. The estimated value of this risk ranged from 0.178 to 0.383. The highest value of 0.383 was noted at measuring point 112, while the lowest was at 23. Furthermore, the estimated values were less than unity or one, as suggested by *UNSCEAR*^1^. However, compared with the international reference value's internal hazard index, it was observed that the estimated values were minor in comparison, suggesting that the impact of the radionuclides emanating from the study area will be more indoor compared to outdoor.

#### Measured gamma dose-rates, (D_rate_)

The gamma dose rates measured are shown in Table 2. The measured values ranged between 54.283 ± 0.78 and 117.531 ± 1.14 nGyh^−1^. The lowest is noted at measuring point 21 and the highest value at measuring location 112, with the estimated mean value of 84.770 ± 0.97 nGyh^−1^. However, high values of measured gamma dose rates above the recommended limit value were observed across the measuring locations and the mean value estimated. Higher values of measured dose rates ranging from 100 nGyh^−1^ above were observed at measuring locations 12, 18, 28, 52, 53, 59, 63, 71, 74, 85, 87, 94, 109, 117, 119 and 120. These locations represent the region of the hotspot for the dose rates. Also**,** the measured values of the radionuclides were compared with the geology of the study area. The result showed that the study area might be rich in coarse-grained inorganic rocks, sandstones, and non-detrital siliceous sediments due to three minerals K-feldspar, K-mica and glauconite^39,40^. This shows that there is the possibility of a high concentration of radionuclides in the study area, especially Potassium-40 (^40^K).

Radiological parameters estimated were correlated with each other. The increasing values of the radiological parameter measured were in the following order: radium equivalent (Req) ˃ gamma dose-rates (D_rate_) ˃ internal hazard (H_int_) ˃ external hazard (H_ext_). As a result of this variation in both H_int_ and H_ext_, the study suggests that the impact of radioactivity measured will be more inside than outside.

Generally, it was observed from Table 3 that the measured natural radioactivity was in increasing order (^238^U < ^232^Th < ^40^K), with the highest concentration being ^40^K. Furthermore, in Figs. 3, 4 and 5, it was noticed that the hotspot region for 238U, 232Th and 40K was observed to be situated in the eastern part of the study area. In terms of radiological parameters, radium equivalent (Req) is within the range of reference value of 370 Bqkg^−1^. In addition, estimated internal hazard (H_int_) has higher values than external hazard (H_ext_), as shown in their calculated mean value (Table 3). Also, the measured gamma dose rate was above the reference value of 59 nGyh^−1^, and This was compared with a similar study carried out by^27^ on soil samples of the study area. The observation showed that it correlates, suggesting that the possible sources of radionuclides in the study area may be associated with the natural deposit of kaolin and gypsum.

**Table 3:** Summary of statistic tools used in this study.

Statistics tools	Measured natural radioactivity			Radiological parameters			Measured gamma dose
	^238^U (Bqkg^−1^)	^232^Th (Bqkg^−1^)	^40^K (Bqkg^−1^)	R_eq_ (Bqkg^−1^)	H_int_	H_ext_	D_rate_ (nGyh^−1^)
Mean	35.44 $\pm$ 0.97	92.57 $\pm$ 1.17	137.59 $\pm$ 2.42	202.15	0.671	0.275	84.770 ± 0.97
Minimum	10.81 $\pm$ 0.69	28.42 $\pm$ 1.12	31.30 ± 1.32	66.00	0.232	0.178	54.283 ± 0.78
Maximum	46.31 $\pm$ 1.43	69.43 $\pm$ 1.76	328.65 ± 2.32	141.76	0.450	0.383	117.531 ± 1.14
UNSCEAR, 2000	33	45	420	370	1	1	59
Standard Deviation	6.57 $\pm$ 0.59	6.93 $\pm$ 0.63	70.10 $\pm$ 6.40				

## Conclusions

In-situ assessment of naturally occurring radiation level in the coastal environments has been carried out using the ground radiometric technique. Radionuclides such as ^238^U, ^232^Th, and ^40^K and gamma dose rates were measured. The result showed that the measured value of ^238^U ranged between 10.81 $\pm$ 0.69 Bqkg^−1^ and 46.31 $\pm$ 1.43 Bqkg^−1^. The mean value was estimated to be 35.44 $\pm$ 0.97 Bqkg^−1^ compared with the world average of 33 Bqkg^−1^. The observation showed that the estimated mean weight of uranium was high when compared with the world average. The measured ^232^Th ranges from 28.42 $\pm$ 1.12 to 69.43 $\pm$ 1.76 Bqkg^−1^ with the calculated mean value of 92.57 $\pm$ 1.17 Bqkg^−1^.

Furthermore, the estimated mean value was compared with the world average of 45 Bqkg^−1^. It was noticed that the estimated mean value was higher. The measured value of Potassium-40 (^40^K) ranged between 31.30 ± 1.32 and 328.65 ± 2.32 Bqkg^−1^ with the estimated mean and standard deviation values of 137.59 $\pm$ 2.42 and 70.10 $\pm$ 6.40 Bqkg^−1^ respectively. In terms of radiological parameters, radium equivalent (Req) is within the range of reference value of 370 Bqkg^−1^. In addition, estimated internal hazard (H_int_) has higher values than external hazard (H_ext_), as shown in their calculated mean value (Table 3). Also, the measured gamma dose rate was noted to be above the reference value of 59 nGyh^−1^. Radiological parameters estimated were also correlated with each other.

The results are in the following order: radium equivalent (Req) ˃ gamma dose-rates (D_rate_) ˃ internal hazard (H_int_) ˃ external hazard (H_ext_). In addition, the results were compared with the geology of the area. The result showed that the location might be rich in coarse-grained inorganic rocks, sandstones, and non-detrital siliceous sediments, radioactive. Therefore, the site may not be safe for residents due to the deposition of anthropogenic radioactive materials and the natural deposit of kaolin and gypsum in the study area. This suggests that gamma radiation monitoring should always be carried out in the study area before embarking on a new building project, whether for office, industry and other uses such as residential, educational institution etc. This is to ensure the area's safety and radioactive content in building materials used. Also, natural ventilation should be used where there are existing buildings to avoid the long accumulation of impact of radionuclides on the residents, which may cause lung cancer.
